# Reuse of Coarse Aggregates Recovered from Demolished Concrete Through the Jigging Concentration Process in New Concrete Formulations

**DOI:** 10.3390/ma18184310

**Published:** 2025-09-15

**Authors:** Artur Bressanelli Teixeira, Carlos Hoffmann Sampaio, Josep Oliva Moncunill, Monica Mariana Davila Lima, Grethel Tamara Herrera La Rosa, Moacir Medeiros Veras, Weslei Monteiro Ambrós, Bogdan Grigore Cazacliu, Albert Solsona

**Affiliations:** 1 Departament d’Enginyeria Minera, Industrial i TIC, Escola Politècnica Superior d’Enginyeria de Manresa, Universitat Politècnica de Catalunya, Av. Bases de Manresa 61-63, 08242 Manresa, Spain; carlos.hoffmann@upc.edu (C.H.S.); josep.oliva@upc.edu (J.O.M.); monica.mariana.davila@estudiantat.upc.edu (M.M.D.L.); grethel.tamara.herrera@estudiantat.upc.edu (G.T.H.L.R.); moacir.medeiros@upc.edu (M.M.V.); 2 Mineral Processing Laboratory, Federal University of Rio Grande do Sul, Porto Alegre 91501-970, Brazil; weslei.ambros@ufrgs.br; 3 MAST GPEM, Université Gustave Eiffel, F-44344 Bouguenais, France; bogdan.cazacliu@univ-eiffel.fr; 4 Departament de Medi Ambient, Romà Infraestructures I Serveis Sau, Av. Dr. Garcia Teixidó, 116, 25242 Miralcamp, Spain; asolsona@romainfraestructures.com

**Keywords:** construction and demolition waste (CDW), concentration, jigging process, concrete, environmental

## Abstract

Construction and demolition waste (CDW) is the most significant portion of solid waste generated throughout the European Union (EU). CDW represents more than a third of the waste generated, considering the waste generated by all economic activities and household waste. The central reuse of CDW is as a base for roads, and in specific cases, it can be reused as recycled coarse aggregates (RA) in the manufacture of precast concrete, new building blocks, bricks, and as RA on new concrete formulations, among other activities. This work aims to enable the concentration of the aggregates mixed in the CDW with the jigging process. The recovered RA was replaced in the concrete, and four different replacement levels (25%, 50%, 75%, and 100%) were analyzed for reuse in new C30/40 concretes. Physical characterization of the material was performed, and compressive strength tests were conducted to verify the RA replacement’s influence on the concrete. The work tests allowed us to observe the positive variation of the material’s physical properties according to the jigging processing and the efficiency of recovering the aggregates. After analyzing the results obtained in the strength force tests, it is possible to conclude that the RA generated can be a substitute for natural aggregates (NA) in new C30/40 concrete formulations. When 100% RJA is used as a replacement, the 28-day compressive strength reaches 33.2 MPa, which is only 6% lower than that of the NA group, reducing the environmental liabilities inherent in the aggregate mining process and generating an economically viable material.

## 1. Introduction

Waste management is one of the most significant challenges of the twenty-first century in the academic sector. Among the many types of solid waste generated, construction and demolition waste (CDW) stands out due to its considerable weight and volume generated within the countries of the European Union (EU) and worldwide. The European Commission constantly monitors the waste generated and the destination each is given. According to research made in 2020 by the European Commission [1], more than 2153 million tons of solid waste are generated annually, accounting for all economic activities and households. The concern with CDW becomes evident when analyzing the percentages of waste generated by each economic activity, where more than 37% of the waste generated comes from the construction sector [2,3].

CDW comprises more than 75% inert material, including concrete, ceramics, bricks, gypsum, and masonry. However, due to the different forms of construction, CDW tends to be very heterogeneous and may contain other materials, such as large volumes of wood, plasterboard, excavated material, etc. [4]. The heterogeneity of what is known as CDW is the main challenge regarding standardization and the definition of a standard route for processing and recovering the material.

Driven by the demands generated, several researchers [5,6,7,8,9,10] have conducted work to analyze economic and environmental aspects, process viability, and other factors involving CDW, such as carbon footprint and the reuse of materials present in CDW. Coelho and De Brito [5,6,7,8] systematically analyzed the implementation of CDW recycling plants. The authors confirmed the economic, environmental, technological, and energetic viability in scenarios with different material inputs, temporal scenarios, and processes. Reis et al. [11], in his research, demonstrated the current uses of recycled CDW in industry, such as the production of sand, the manufacturing of concrete blocks, the reuse in highway bases, directly in the concrete mix, and even in use as adsorbents in the treatment of liquid effluents. The researchers demonstrate the possibility of reusing the material, although the work warns about the need for parameterization and concentration of materials to avoid contaminants. Different authors [11,12,13,14,15] warned about the restriction on the use of recycled CDW products due to the presence of pollutants, which can affect the characteristics of the materials, as well as durability, resistance, workability, etc.

The presence of contaminants negatively influences the direct properties of the aggregates generated. In the research made by De Brito et al. [16], it was explained that the presence of bricks mixed in the recycled aggregates can render the CDW unusable for direct reuse as aggregates in the cement mix. Contaminants can reduce the inherent strength of the concrete formulation, where it contains such contaminants. The contaminants negatively influence the fundamental properties of the aggregates, such as the size distribution, shape index, bulk density, and water absorption.

The current CDW processing method is simple and aims to generate recycled aggregates for reuse in street and highway subbases. CDW processing consists of separating (manually or using machinery) large or low-density materials (paper, glass, plastics, wood, etc.) mixed with materials such as paper, wood, glass, and plastic parts. Subsequently, the inert part (bricks, concrete, gypsum, ceramics, etc.) is comminuted to separate the fine part (<5 mm) and generate the inert coarse aggregates (<20 mm and >5 mm) [10,17,18]. New processing routes have been studied [18,19,20,21,22] to generate new products with added value, such as recovering aggregates present in CDW and reusing them in new concrete formulations.

Techniques were created to analyze the reuse of CDW to avoid generating mixed CDW material with contaminants, such as bricks, gypsum, ceramics, etc. CDW pre-selection techniques at the site of its generation, separating the concrete from other materials, allow the generation of recycled concrete aggregates (RCA), a material composed only of concrete. The RCA generation facilitates the parameterization of a process where concrete is recycled, enabling the recovery of aggregates present in the material with a low impurity content [23,24,25]. RCA is composed of coarse aggregates (<20 mm and >5 mm), fine aggregates (<5 mm), and cement paste (contaminant in the aggregate reuse process). Therefore, during the recycling process, as demonstrated, the fine portion of the cement paste material is separated, generating a coarse material composed of fully liberated aggregates and aggregates with a layer of adhered cement paste.

Salgado and Silva [26], in the investigation about replacing RCA in new concrete, concluded that recycled aggregates are more porous and have a more complex microstructure than natural aggregates. This higher porosity acts as a presumable canal for water transport and aggressive agents such as chloride ions, affecting the material’s durability. Because of the higher water absorption, recycled coarse aggregates typically need more water than conventional concrete to obtain the same workability; it also affects the homogeneity of the fresh concrete during casting, reducing the mechanical strength of the concrete.

Some studies pointed out that the reduction in compressive strength (CS) was between 12 and 25% when 25–30% [27] or 100% NA was replaced by CDW aggregate, as the study of Li et al. [28]. Ignjatovic et al. [29] studied the flexural behavior of the concrete when RCA is added to the mix. When replacing 50% of the natural aggregates present in concrete with RCA, a reduction of 7.8% in CS was observed, and when 100% replacement was analyzed, a decrease of 4.2% in CS was observed. The study’s results demonstrate the direct relationship between the replacement of NA by RCA and the reduction of CS of concrete.

To reduce the influence of material replacement, the need to reduce the volume of contaminants (cement paste) in the RCA is evident. Several studies [18,19,20,30] have been carried out to define beneficiation routes to recover the aggregates present in the RCA with the lowest possible cement paste content, thus allowing their reuse in new concrete formulations. One of the most promising research processes for the concentration of aggregates in CDW is the jig process [15,19,21,31,32,33,34,35]. The research has shown the technical feasibility of the CDW contaminant removal process with an efficiency above 99%. The jigging process is robust and low-cost, thus allowing the economic viability of the jigging process to benefit CDW. An analysis of the European scenario shows the possibilities of CDW management and other aspects [36]. This work will use the jigging process to concentrate the aggregates present in the RCA and to attest to the quality of the material generated by comparing the observed compressive strength values when replacing the NA with RCA and JRA (aggregates recovered through the jigging process).

### Jigging Process

Jigging is one of the oldest gravity concentration processes ever used by humanity. According to Lyman [37], the basic principles of the process date back to Ancient Egypt. The texts written by Agricola in 1556 [38] show that the process was already being used routinely and has since been developing for industrial applications.

**Figure 1:**
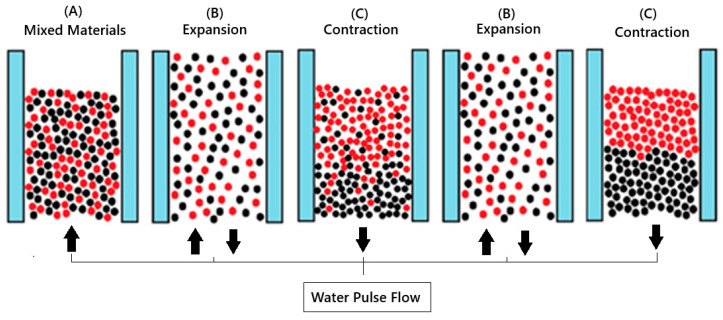
Model representing particle movement within the jig chamber. Red particles represent lighter material, and black particles represent denser particles. Image divided into three stages according to the water flow at each stage of the process. (A) Upward flow, with all the mixed material representing a unit mass volume. (B) Material expansion, fluidization of the bed, allowing particle separation. (C) Downward water flow, material contraction, and stratification of the jig bed. Adapted from Teixeira et al. [19].

Jigging is a hydraulic separation process that consists of the repeated vertical expansion (dilation) and contraction (compression) of a bed of particles by the pulsating movement of a fluid (water or air). The result is the stratification of the bed, which corresponds to the separation of particles into layers or strata of increasing densities from the top to the bottom [31]. Sampaio et al. [31] explain that although the jig in the air has the advantage of not using water in the separation process, it has a lower efficiency. The fluidization of the jig bed allows materials of similar densities, sizes, and shapes to stratify at the top or bottom of the jig according to their characteristics. The material stratifies according to the action of the force of gravity that acts on the particle, allowing separation mainly by densities into layers. The dense material concentrates at the bottom of the jig bed due to its differential sedimentation velocity, and the lighter material is separated at the top of the jig bed [33]. Figure 1 shows the process of expansion and dilation of the jig bed during material processing.

One of the leading researchers of the jigging process, F.W. Mayer [38,39], proposed a theory to explain the stratification in the jigging process called the “Potential Energy Theory of Jigging”. Mayer suggests a difference in gravitational potential energy between the thoroughly mixed and stratified states of materials concerning densities and that the relationship between the potential energies between the systems is responsible for the stratification in the jigging process.

Considering the characteristics of RCA, where the mixture between aggregates (material with higher density) and cement paste (material with lower density due to its porosity and chemical composition) is observed, the jigging process tends to show itself as a promising method of concentrating the aggregates present in RCA. The process enables the removal of the cement paste mixed with the material and generates a high-purity aggregate that can be reused in new concrete formulations. This article uses the jigging process to concentrate aggregates from RCA and develop material with low cement paste content and characteristics similar to natural aggregates typically used in concrete (RJA—recycled jigging aggregates). After the jigging process, concrete specimens were generated with NA, RCA, and RJA in different replacing ratios, comparing the results of the materials and enabling the generation of a new route for RCA beneficiation and recovery of coarse aggregates present in CDW.

## 2. Materials and Methods

### 2.1. Material Preparation

In this work, three different materials were used. Conventional natural aggregate (NA) was defined as the reference for physical characteristics and compressive strength tests. Recycled concrete aggregates (RCA) were analyzed to determine the characteristics of the material typically generated in CDW concentration plants. The measured values were compared with jigged recycled aggregates (RJA), material obtained from the processing of RCA with the jigging process.

**Figure 2:**
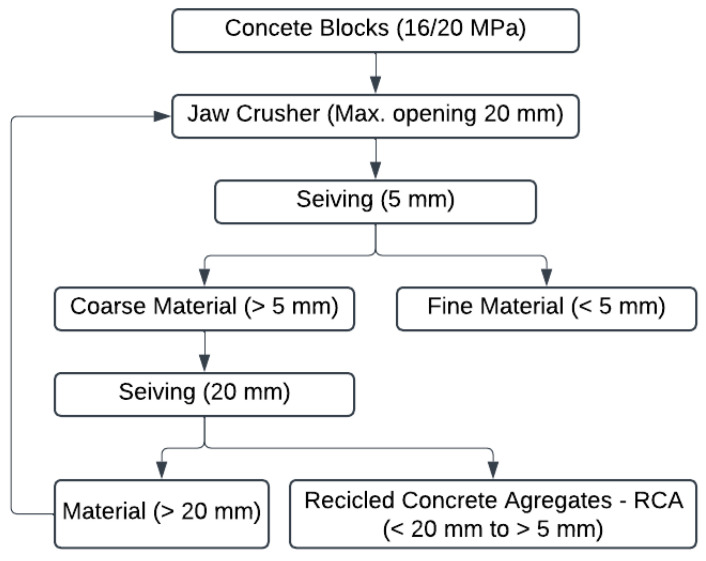
Flowchart of the processes used to generate the RCA.

Conventional structural concretes (C16/20 MPa) were used to generate the RCA used in the tests, as demonstrated in Figure 2. The material was subjected to a primary comminution process in a jaw crusher configured with a maximum particle size opening of 20 mm. The material was sieved to a 5 to 20 mm range to emulate the beneficiation process typically used in CDW concentration plants, generating the RCA. The finer material (<5 mm) was not used in the present study. Table 1 summarizes the three materials that will be analyzed and are part of the present study.

### 2.2. Jig Concentration Process

#### 2.2.1. Jig Equipment

**Figure 3:**
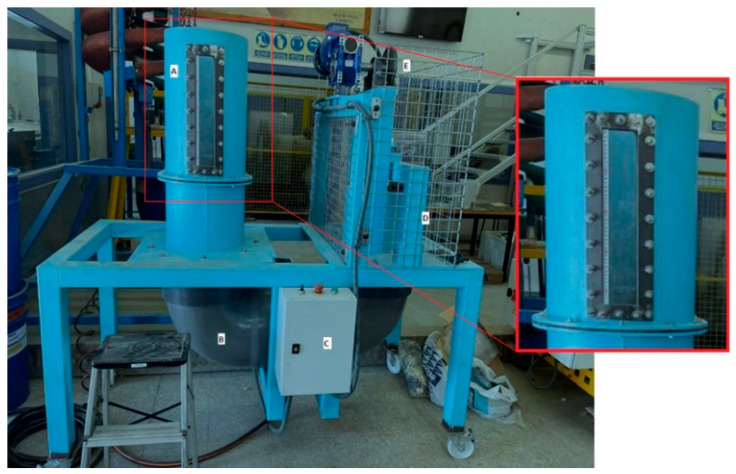
Jig equipment used in the experiments: (**A**) Jig chamber (enlarged), (**B**) water passage duct, (**C**) electric panel, (**D**) pump chamber, (**E**) motor. Adapted from Teixeira et al. [19].

Material concentration tests were performed on a laboratory-scale water jig. Figure 3 shows the jigging machine used in the present study. The material is manually introduced into the jig from the upper part of the jig chamber (A), where the bed fluidizes and the material stratifies (as shown in Figure 1). During the test, the piston (D) drives the water to the chamber through the duct (B). After each test, the material is manually removed from the top of the jig chamber according to a previous analysis of the layers. It is placed to dry in an oven at 70 °C for 12 h to quantify the removed portions and direct them to the characterization processes. The equipment works in batches.

After preliminary tests of the jig’s efficiency and suitability for the concentration of RCA, a frequency of 35 pulses per minute was defined, a piston movement amplitude of 14 cm, and a 3-min retention time for the material until stratification was achieved.

#### 2.2.2. Jigging Process

**Figure 4:**
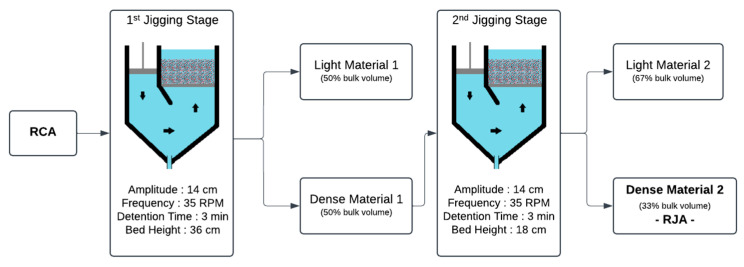
The flowchart of the two-stage jigging processes is used to concentrate the RCA and generate RJA. Adapted from Teixeira et al. [19].

Figure 4 shows the flowchart of the two-stage jigging process to which the RCA was subjected to generate the RJA. In the first jigging stage, two different materials were generated. The lighter material, representing 50% of the apparent volume of the RCA inserted into the jig chamber, was discarded, and the denser material, representing the remaining 50% of the volume of the jig chamber, was directed to the second jigging stage. In the second stage, the material was subjected to another concentration process, generating two different materials: a lighter material not used in this work, comprising 67% of the bulk volume from the second jigging stage, and a denser material (RJA) representing 33% of the volume from the second jigging stage. During the first stage of jigging, a bed height of 36 cm was used, while in the second stage, a bed height of 18 cm was used. The tests were performed in duplicate to analyze the reliability of the proposed flowchart.

### 2.3. Characterization Analysis

#### 2.3.1. Densimetric Distribution Test and Analysis of the Concrete Substrate Constitution

Due to the presence of particles formed only by cement paste (lower densities), particles mixed with cement paste and aggregates (intermediate densities), and completely liberated aggregates (higher densities), an analysis of the densimetric distribution of the NA, RCA, and RJA used in the present study was carried out. The following granulometric ranges were analyzed: ρ < 2.4 g/cm^3^, 2.4 < ρ < 2.5 g/cm^3^, 2.5 < ρ < 2.55 g/cm^3^, 2.55 < ρ < 2.6 g/cm^3^, 2.6 < ρ < 2.65 g/cm^3^, 2.65 < ρ < 2.7 g/cm^3^, 2.7 < ρ < 2.75 g/cm^3^, and 2.75 < ρ < 2.8 g/cm^3^. Sink and float tests were conducted, and the dense media used for analysis were generated with a sodium polytungstate solution. The density of the solutions was measured with a manual Density Meter (DMA 35—Anton Paar GmbH, Graz, Austria).

To analyze the composition of the particles in each granulometric range, the materials obtained were subjected to chemical digestion according to the method proposed by Akbarnezhad et al. [40]. The material is weighed and submerged in a solution of sulfuric acid at a concentration of 3 molar for 8 h. The process is repeated until, after visual analysis, only aggregates are present, and there is no more cement paste adhered to the materials. After digestion, the material is weighed. The difference in weight is taken as the cement paste present, and it is sieved to 5 mm to define the presence of coarse aggregates (>5 mm) and fine aggregates (<5 mm). This analysis determines which portion of the material has the highest concentration of coarse aggregates and can be concentrated with the jigging process.

#### 2.3.2. Specific Density (OD), Saturated Specific Density (SSD), Bulk Density, and Water Absorption

Understanding that RCA is composed of aggregates and cement paste (porous material, which changes the characteristics and modifies the water absorption of the material), an analysis of the physical characteristics of the material is necessary in its dry form (OD), as well as in its water-saturated form (SSD). ASTM C-127-07 [41] was used to perform the analyses, and details of the experimental procedure, as well as data processing, are set out in the standard. During the tests, specific density (OD), saturated specific density (SSD), bulk density, and water absorption were defined.

The equations for defining the measured values are:

(1) Water Absorption (%) − [(B − A)/A] × 100

(2) $$\mathrm{Bulk}\;\mathrm{Density}\;(\mathrm{kg}/m^{3})-997.5\;A/(A-C)$$

(3) $$\mathrm{Specific}\;\mathrm{Density}\;(\mathrm{OD})\;(\mathrm{kg}/m^{3})-997.5\;A/(B-C)$$

(4) $$\mathrm{Specific}\;\mathrm{Density}\;(\mathrm{SSD})\;(\mathrm{kg}/m^{3})-997.5\;B/(B-C)$$

where

A = mass of oven-dry test sample in air, g;

B = mass of saturated–surface–dry test sample in air, g;

C = apparent mass of saturated test sample in water, g.

#### 2.3.3. Form Factor

Due to the randomness of the fractures that occur in the concrete during the comminution process, particles with different spatial conformations are formed, which can be more lamellar or spherical. Such characteristics directly influence the bulk density of the material and modify the kinetics of concentration of the material inside the jig.

EN 933-4:2008 [42] was used for particle shape factor analysis. The standard defines the selection of 200 particles according to the granulometric stratification and the measurement with a scale of the longest and narrowest dimensions of the material. The shape factor value is the average ratio of each selected particle’s most extensive and diminutive dimensions.

### 2.4. Concrete Production and Compressive Strength Test Parameters

Test specimens of C30/40 concrete were prepared with each of the materials to analyze the mechanical behavior of the concrete in compressive strength tests. Table 2 shows the mixing proportion that was used in the concrete. Test specimens were manufactured according to the EHE 08 [43] normative, with dimensions of 15 × 15 × 15, and subjected to the tests. CEM II/B-M (P-LL) 42.5 R type cement was used, with a minimum compressive strength of 42.5 MPa at 28 days. No additives were used.

Quartz sand with a maximum size of 5 mm was used to produce the test specimens as fine aggregates. For comparison and correlation of the replacement of natural aggregates by recycled aggregates, a reference specimen made only with natural aggregates was manufactured. In contrast, for the specimens containing recycled aggregates, the natural aggregates were replaced by contents of 25, 50, 75, and 100% by recycled aggregates (RCA and RJA), as shown in Table 3. The aggregates used to manufacture the analysis specimens were the same type as those present in the RCA that was introduced into the jig. Aggregates from crushed gravel were used.

The compression strength tests were conducted in a laboratory with a controlled temperature of 20 °C ± 2, with an uncertainty of 1% in the measurements performed. The tests were carried out after 7 and 28 days, according to the dictum in UNE-EN 12390-3:2009 + AC1:2011 [44]. The 28-day curing process was conducted in the same conditions as mentioned. The standards state the performance of duplicate tests and reliability analysis.

## 3. Results and Discussion

### 3.1. Concrete Substrate Analysis and Densimetric Distribution of the Recycled Concrete Aggregate (RCA)

Sink-float tests were performed to understand the densimetric profile of the RCA used in the present work. Table 4 shows the results obtained from the densimetric stratification of the material with a particle size range of 5 to 20 mm.

**Figure 5:**
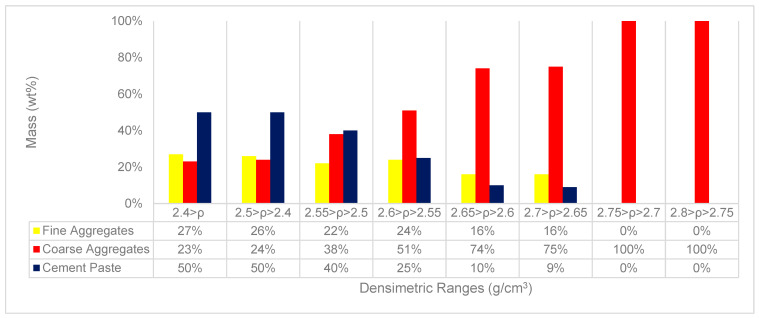
The content of cement paste, coarse aggregates, and fine aggregates in the different RCA densimetric fractions (ρ < 2.4 g/cm^3^, 2.4 < ρ < 2.5 g/cm^3^, 2.5 < ρ < 2.55 g/cm^3^, 2.55 < ρ < 2.6 g/cm^3^, 2.6 < ρ < 2.65 g/cm^3^, 2.65 < ρ < 2.7 g/cm^3^, 2.7 < ρ < 2.75 g/cm^3^, and 2.75 < ρ < 2.8 g/cm^3^) was measured—material with a 5 to 20 mm granulometric range. Adapted from Teixeira et al. [19].

From the densimetric analysis performed on the material, it is possible to observe a large densimetric variation, which implies the presence of materials with different characteristics and compositions. Particles with a density below 2.6 g/cm^3^ are formed by fine aggregates mixed with the cement paste or by coarse aggregates with a layer of cement paste adhered (Figure 5). The cement paste and fine aggregates do not present a measurable degree of liberation due to their complete mixing. A considerable portion of this material is removed from the RCA during the comminution process.

Cement paste adhered to the aggregate particles negatively modifies several physical characteristics compared to natural aggregates, such as water absorption, saturated and oven-dried specific gravity, and the material’s porosity. Such influence can make the material unsuitable for reuse as aggregates in new concrete formulations. Given the difference in strength between the cement paste and the aggregates present, concrete tends to fracture at the interface of the materials, the so-called interstitial zones (ITZ). Fractures in the ITZ allow the formation of particles with completely liberated aggregates [14].

Analyzing the possible variation in the composition of the particles in the RCA, the material was dissolved in acid (as mentioned in item 1.3.1), thus defining which portions of the material have a higher coarse aggregate ratio and are plausible to concentrate. Figure 5 shows the graph with the contents of each RCA component material (fine aggregates, coarse aggregates, and cement paste).

The composition of the analyzed densimetric fractions presented values as expected, similar to the Sampaio et al. results [45]. After visual analysis, the material with a density above 2.75 g/cm^3^ was considered completely liberated coarse aggregates, representing 4% of the total volume of the RCA.

Materials with a density above 2.65 g/cm^3^ presented a high content of coarse aggregates and a low ratio between fine aggregates mixed with the cement paste and the coarse aggregates present. This material represents 34% of the particles in the RCA and can be concentrated to generate the RJA—a material with characteristics closer to the NA used in the construction sector.

### 3.2. RCA Processing, RJA Generation—Jig Concentration Process

**Figure 6:**
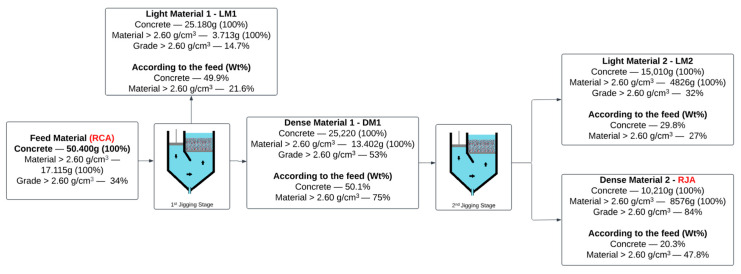
The flowchart of the jigging process concentrates the aggregates present in the RCA and generates the RJA. Adapted from Teixeira et al. [19].

It is worth remembering that the jigging process concentrates particles based on the difference in density between the materials. The jigging tests conducted in this work were designed to concentrate material with a density greater than 2.6 g/cm^3^, which, as shown in Item 2.1, represents 34% of the RCA introduced in the jigging process. Figure 6 shows the flowchart of the jigging process used to concentrate RCA and generate RJA after two stages. The figure shows the weight of the materials inserted and generated in each jigging stage, the content of particles with a density above 2.6 g/cm^3^, and their recovery rate compared to the material inserted in the jigging system.

The RCA introduced in the jigging process contains 34% of the material with a density greater than 2.6 g/cm^3^. After the first jigging stage and the stratification of the RCA, the “Light Material 1—LM1”, which makes up 50% of the apparent volume, has a content of 14% dense material and is composed mainly of cement paste thoroughly mixed with fine aggregates. The presence of dense material is explained by Sampaio and Tavares [31] and Teixeira et al. [19], who explain the entrapment of dense material in upper layers due to the impossibility of percolation due to the interaction with other particles, buoyancy force, and particle shape of the material as the jigging process takes place. Due to its dense material content, LM1 can be recirculated to concentrate this fraction.

The other 50% of the apparent volume of the first jigging stage is composed of “Dense Material 1—DM1”. With the concentration of the densest material in the lower layers, DM1 has an increased content of 53% of the particles with a density above 2.6 g/cm^3^, recovering more than 75% of the dense material in the RCA introduced into the feed. The pre-concentrate DM1 was redirected to the second jigging stage to increase aggregate concentration and generate the RJA.

DM1 reprocessing was performed by stratifying the material into two layers. The upper layer, known as “Light Material 2—LM2”, is composed of 66% of the apparent volume of DM1 and has a 34% dense particle (density > 2.6 g/cm^3^) content. LM2 has characteristics similar to those of RCA introduced in the feed, and within a closed aggregate recovery circuit, this material is recirculated to recover it. LM1 and LM2 were not used in the present work. Composed of 34% of the apparent volume from DM1 introduced in the second jigging stage, “Dense Material 2—RJA” has a content of 84% of dense particles and concentrates 47.8% of the thick material that was introduced in the feed as RCA in the first jigging stage. RJA comprises completely liberated aggregates with a thin layer of cement paste, eliminating the light particles of cement paste and fine aggregates. The material represents 20.3% of the weight of the RCA that was inserted into the feed, thus enabling a reduction of more than 20% in CDW disposal in a reuse and recycling scenario.

### 3.3. Physical Properties Analysis

After the proposed jigging process, the materials were sent for densimetric and physical properties analyses, as explained in Item 1.3. The comparison between the material properties allows the observation of the jigging process’s influence on the material’s characteristics. It will enable the comparison between the materials and the adequacy of the RCA to generate a material with characteristics closer to NA. Table 5 shows the values of specific density (OD), saturated specific density (SSD), bulk density, water absorption, and form factor measured in the three materials studied (NA, RCA, and RJA) and compares them with the data obtained by Salgado [46].

Table 5 shows the test values of the characteristics that most influence aggregates when inserted in the manufacture of concrete. The comparison with the values observed by Salgado [45] shows very similar values and behaviors of the analyzed parameters and corroborates the possibility of inserting RJA as aggregates in concrete. Density is one of the fundamental parameters of aggregates and is essential to designing concrete mixes and controlling several properties of the resulting concrete. When analyzing the evolution of properties, it is possible to correlate a direct relationship between the cement paste content present in the material and the measured values. Teixeira et al. [19] studied the relationship between cement paste contents and directly related properties. The authors concluded that increasing cement paste content negatively influences water absorption and dry and saturated densities. Due to its high porosity, the cement paste can modify the hydration dynamics of the concrete and the volume of water required for manufacturing, influencing the characteristics of the concrete in its fresh and hardened state. Limbachiya et al. [47] also conducted studies with RCA with different contents of adhered cement paste. With the increase in the cement paste content, an increase in water absorption and a reduction in the density values obtained were observed.

The bulk density values observed in the present study (ranging from 1.31 to 1.41 g/cm^3^) are consistent with those reported by Brito and Saikia [14], where the authors state that they are generally in a range of 1.15 to 1.4 g/cm^3^. Ferreira et al. [48] studied pre-saturation techniques for recycled aggregates in concrete manufacturing. They stated that the negative variation in the bulk density values of RCA compared to NA is due to the volume of voids in the material. Sampaio et al. [45] explain that the randomness of the fracture of concrete blocks, when subjected to the comminution process, allows the formation of particles with random dimensions, influencing the formation of voids.

In the work by De Juan [49], the author reported values of 0–4% of water absorption for NA; however, when analyzing aggregates with adhered cement paste, the values can reach 16–17%. Table 5 shows a reduction of 3.53% of the water absorption values when comparing the values measured for RCA (4.73% of water absorption) with RJA (1.2% of water absorption). Correlating the parameters and verifying a reduction of more than 30% of the cement paste content is possible. Given the significant variation in water absorption, Santos et al. [50] proposed a method of pre-saturation of RA to standardize the volume of water necessary for manufacturing concrete.

**Figure 7:**
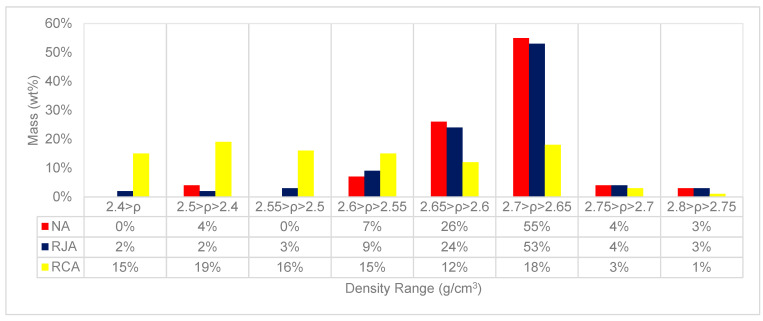
Comparative graph of the densimetric distribution of NA, RJA, and RCA.

From the understanding generated from Table 5, where the physical characteristics of the material generated after the jigging process (RJA) are similar to those found in the NA studied in the present work by Salgado [46], an analysis of the densimetric stratification of the materials is necessary to confirm the densimetric strata and their correlations with the cement paste and aggregate contents. Figure 7 demonstrates the densimetric distribution of RCA, NA, and RJA. Figure 7 shows the comparative graph between the densimetric stratifications observed in the NA, RJA, and RCA materials.

Despite the homogeneous densimetric distribution of the RCA, where the particles are spread throughout the granulometric range when analyzing the values obtained in the RJA, it is possible to observe a considerable reduction in the content of particles with densities below 2.6 g/cm^3^. The observation allows us to state that the jigging process was efficient in removing the lighter particles present in the RCA and enabled the generation of a material (RJA) with characteristics similar to those of the NA. Analyzing the densimetric stratifications of the NA and RJA, it is possible to observe more than 75% of the particles in a densimetric range that varies from 2.65–2.70 g/cm^3^; these particles are composed of aggregates with a fine cement paste, as demonstrated in Figure 5.

### 3.4. Compressive Strength Tests

**Figure 8:**
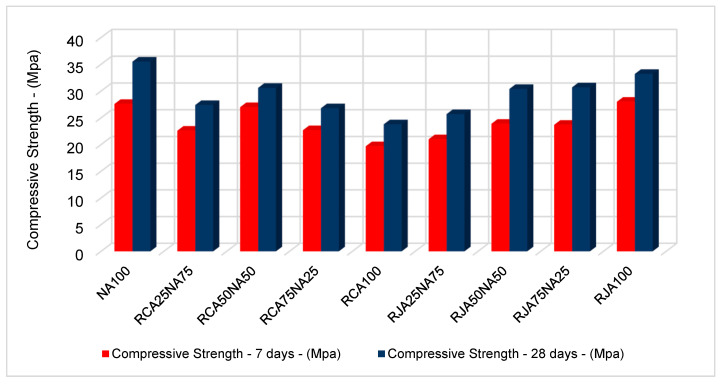
Summary of the results of compression strength tests performed at 7 and 28 days.

Compressive strength tests in C30/40 concretes were performed and measured at 7 and 28 days of wet curing to understand the influence of replacing natural aggregates with recycled aggregates. Concrete mixes with 25, 50, 75, and 100% replacement of natural aggregates by RJA and RCA were analyzed. Table 6 shows the maximum values of compression strength found on the tests manufactured with the materials studied in the present work. A test was carried out with concrete manufactured with 100% natural aggregates to define a reference value. The sample’s nomenclature and mixing proportions are listed in Table 3. Figure 8 summarizes the values obtained from the compression strength test for each proposed mixture at 7 and 28 days.

Regarding the results obtained in the compression strength tests, it is possible to observe a reduction in the concrete strength as RCA is added. When comparing the values measured in the control concrete (NA100) and the concrete composed 100% with RCA, it is possible to observe a reduction in the magnitude of 30% in the absolute value of the compression strength. This behavior is explained by Brito and Saikia [14] and Exteberria et al. [27], where the authors demonstrate that the porous composition of the recycled aggregates tends to create more ITZ, causing the concrete to become weaker after drying. Concrete made with 100% coarse recycled aggregate requires a high amount of cement to achieve high compressive strength and, consequently, is not an economic proposition as it is not cost-effective. In Bravo et al. [51] work correlating the aggregate replacement rate with compressive strength, the authors observed a reduction of 5 and 26% in concrete strength when NA was replaced by RCA at levels of 20 and 100%, respectively.

**Figure 9:**
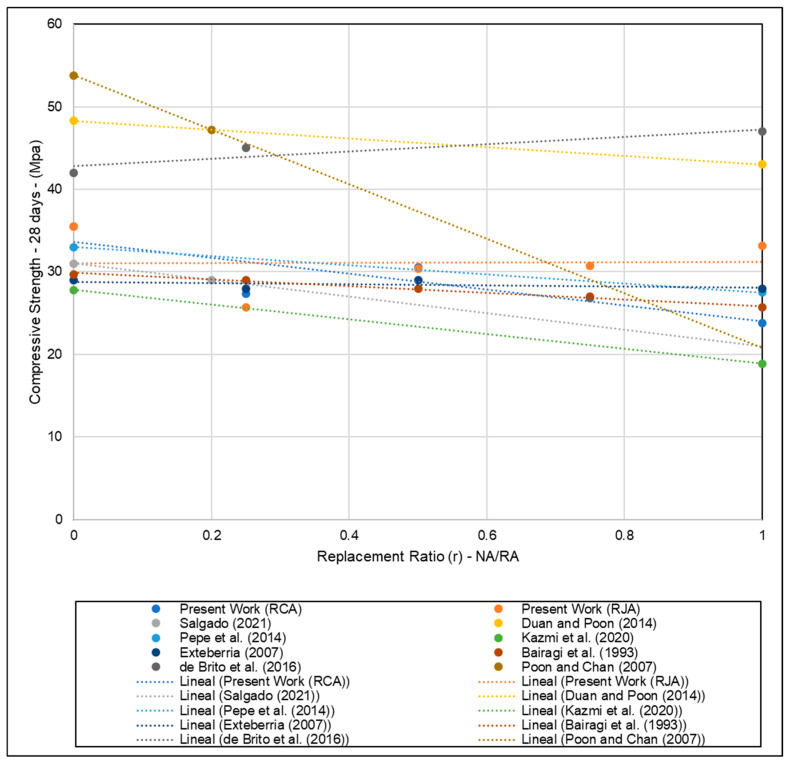
Comparison of the compressive strength of concrete specimens concerning the rate of replacement of NA by RA based on the results obtained by several authors [27,46,52,53,54,55,56,57] and the present work.

Analyzing the evolution of the results obtained for concrete manufactured with RCA as the substitution rate increases, it is possible to observe a variable behavior of the measured compressive strength values, measuring 27.4 MPa, 30.6 MPa, 26.8 MPa, and 23.8 MPa for the replacement of 25, 50, 75, and 100%, respectively. The values obtained are lower than those proposed in manufacturing a C30/40 concrete. The behavior is explained by the randomness of the particles present in the RCA, where the characteristics of the particles, according to the portions of the aggregates used, can vary considerably. Despite the variation in the measured values, analyzing the trend line shown in Figure 9, it is possible to observe a downward trend in the compression strength values as the replacement rate approaches 1.

The results agree with those evaluated by de Brito et al. [52]; as the substitution rate of NA by RA increases, there is a positive variation in the measured compressive strength results by the authors. Figure 9 compares the values obtained in compression tests carried out by several authors [27,46,52,53,54,55,56,57] and shows the trend lines of the materials according to the rate of replacement of natural aggregates by recycled aggregates from different sources and treatment techniques.

Considering the various methods employed to concentrate aggregates present in CDW, Sampaio et al. [58] conducted a study on the economic viability of using different concentration methods (sensor-based sorting (SBS), water jigs, and air jigs) and concluded that the water jig process offered greater financial viability for concentrating coarse aggregates. Due to its robustness, operational simplicity, and extensive industrial knowledge, the water jig process proved to be the most viable among the analyzed processes.

Analyzing the concretes manufactured with the concentrated material from the jigging process, it is also possible to observe a reduction in the compression strength values. However, with the reduction in the cement paste content and consequent similarity of the physical characteristics of the NA with the RJA, when comparing the control concrete (NA100) with the concrete manufactured 100% with RJA (RJA100), a 9% reduction in the compression strength value is observed, presenting an improvement of more than 20% of the strength values observed with the replacement of 100% of the natural aggregates by RCA.

Comparing the results obtained with the replacement of NA by RCA and RJA, it is possible to observe a difference in the strength results. The results are more stable and have values closer to the reference values. Most of them are within the proposed values for C30/40 concretes. Analyzing the trend line of the tests performed with RJA, it is possible to observe linearity, which indicates an adequacy of the material’s characteristics for reuse as aggregates. According to the characterization analyses, RJA has more homogeneous and regular characteristics and a cement paste content 30% lower than RCA. Teixeira et al. [19] demonstrated the direct relationship between the cement paste’s presence and the aggregates’ characteristics. They explained that the cement paste negatively affects the properties and can cause changes in the materials that use RCA without treatment in their composition. As seen in Item 2.3, after processing with the jig, the RJA has characteristics closer to those measured in the NA, corroborating the values obtained in the compression tests.

## 4. Conclusions

The main conclusions of this paper are the following:

(I) The results observed in recycled aggregates, in a granulometric range of 5 to 20 mm, originating from C16/20 concrete, have a large densimetric variability, with a high cement paste content and a negative variation in physical characteristics.
(II) The proposed jigging process efficiently removes less dense particles and can improve RCA’s physical characteristics. The densimetric stratification of the material and concentration of the coarse aggregates present, enabling the generation of a recycled aggregate (RJA) with characteristics similar to those found in natural aggregates.
(III) The reduction in the cement paste content of the material is directly linked to the water absorption of the recycled aggregates when compared to the physical properties of the materials. The cement paste content also negatively influences the behavior of concrete when analyzing the values measured in the compression strength tests.
(IV) A reduction of up to 30% in strength values is observed when the replacement of NA by RCA is analyzed. This presents a considerable variation in values due to the increase in the replacement rate of NA by RCA. The material cannot be used as a substitute for NA in C30/40 concrete formulations.
(V) A 13% and 6% reduction in strength is observed when natural aggregates are replaced by the material obtained after the jigging process (RJA), at 50 and 100%, respectively, after jigging tests.

## Figures and Tables

**Table 1:** Summary of the materials analyzed in this work.

Materials	Description
NA	Natural Aggregates—Aggregates are usually used in the civil industry to manufacture concrete.
RCA	Recycled Concrete Aggregates—Material generated after the comminution and screening (Figure 2), typically generated in CDW concentration plants.
RJA	Recycled Jigging Aggregates—Material generated after the two stages of the jigging process proposed in this work.

**Table 2:** Concrete mix proportions.

Material	Mix (kg/m^3^)	Proportion (%)
Cement	150	15
Water	75	7.5
Coarse Aggregates	525	52.5
Fine Aggregates	250	25
Additives	0	0

**Table 3:** Concrete sample specifications.

Sample Name	Coarse Material Replacement Ratio		
	Natural Aggregates (NA) (%)	Recycled Concrete Aggregates (RCA) (%)	Recycled Jigging Aggregates (RJA) (%)
NA100	100	0	0
RCA25NA75	75	25	0
RCA50NA50	50	50	0
RCA75NA25	25	75	0
RCA100	0	100	0
RJA25NA75	75	0	25
RJA50NA50	50	0	50
RJA75NA25	25	0	75
RJA100	0	0	100

**Table 4:** Densimetric distribution of RCA.

Analysis	Densimetric Ranges (g/cm^3^)							
	2.4 > ρ	2.4 < ρ < 2.5	2.5 < ρ < 2.55	2.55 < ρ < 2.6	2.6 < ρ < 2.65	2.65 < ρ < 2.7	2.7 < ρ < 2.75	2.75 < ρ < 2.8
Retained Material	15%	19%	16%	15%	12%	18%	3%	1%

**Table 5:** Analysis of the physical parameters of the materials used in the present work and their comparison with the data obtained by Salgado [46].

Proprieties	Present Work			Salgado [46]	
	NA	RJA	RCA	NA	RCA
Specific Density (OD) (g/cm^3^)	2.67	2.66	2.59	2.57	2.25
Specific Density (SSD) (g/cm^3^)	2.65	2.60	2.47	2.60	2.39
Bulk Density (g/cm^3^)	1.38	1.38	1.37	1.42	1.31
Water Absorption (%)	0.72	1.20	4.73	1.20	6.40
Form Factor	2.09	2.07	2.19	1.87	1.97
Cement Paste Content (%)	0.00	15.10	46.20	-	-

**Table 6:** Results of maximum values of compressive strength tests performed with concrete mixtures replacing natural aggregates with recycled aggregates.

Concrete Samples	Compressive Strength—7 Days—(MPa)	Compressive Strength—28 Days—(MPa)
NA100	27.6	35.5
RCA25NA75	22.6	27.4
RCA50NA50	27	30.6
RCA75NA25	22.7	26.8
RCA100	19.7	23.8
RJA25NA75	21	25.7
RJA50NA50	23.9	30.4
RJA75NA25	23.7	30.7
RJA100	28	33.2

## Data Availability

The original contributions presented in this study are included in the article. Further inquiries can be directed to the corresponding author.
